# Individuals vary in their overt attention preference for positive images consistently across time and stimulus types

**DOI:** 10.1038/s41598-024-58987-8

**Published:** 2024-04-15

**Authors:** Nitzan Guy, Asael Y. Sklar, Revital Amiaz, Yael Golan, Abigail Livny, Yoni Pertzov

**Affiliations:** 1 Department of Psychology, Hebrew University of Jerusalem, Jerusalem, Israel; 2 Department of Psychology, Tel Aviv University, Tel Aviv, Israel; 3 Arison School of Business, Reichman University, Herzliya, Israel; 4 Faculty of Medical and Health Sciences, Tel Aviv University, Tel Aviv, Israel; 5 Department of Psychiatry, Sheba Medical Center, Tel-Hashomer, Israel; 6 The Diagnostic Neuroimaging Laboratory, Sheba Medical Center, Tel-Hashomer, Israel; 7 Sagol School of Neuroscience, Tel Aviv University, Tel Aviv, Israel

**Keywords:** Positivity preference, Depression, Valence, Individual differences, Eye tracking, Psychology, Human behaviour, Depression

## Abstract

What humans look at strongly determines what they see. We show that individual differences in the tendency to look at positive stimuli are stable across time and across contents, establishing gaze positivity preference as a perceptual trait that determines the amount of positively valence stimuli individuals select for visual processing. Furthermore, we show that patients with major depressive disorder exhibit consistently low positivity preference before treatment. In a subset of patients, we also assessed the positivity preference after two months of treatment in which positivity gaze preference increased to levels similar to healthy individuals. We discuss the possible practical diagnostic applications of these findings, as well as how this general gaze-related trait may influence other behavioral and psychological aspects.

## Introduction

Gaze position is a major gatekeeper for information used by the visual system. Most visual information is captured from a small area around the center of gaze^1^. Thus, where the eyes fixate determines which visual information will be processed in detail, and therefore determines visual experience. Individuals who consistently look at different types of stimuli are expected to experience consistently different environments. Here, we test whether preferentially looking at positive stimuli is a consistent individual trait by examining if it is stable across time and stimuli. Next, we examined the construct validity of positivity preference by testing gaze preference in patients with major depressive disorder (MDD^2^) before and (in a subset of patients) after treatment with selective serotonin reuptake inhibitors (SSRIs).

Over the last decades, a growing number of eye-tracking studies examined the factors involved in guidance of gaze. While earlier eye-tracking studies mostly focused on the influence of stimuli and task factors on gaze behavior^e.g., 3–5^, recent studies considered the observer factor—how individuals differ in scanning the same stimuli under the same task demands. These studies focused mostly on low level features of gaze behavior (e.g., saccade amplitudes^6,7^) and deployment of gaze towards specific semantic features (e.g., fixations on faces or text^8,9^). Here we propose that people differ in their gaze preferences based on a more abstract feature of the stimulus—its affective valence.

Affective valence, whether a stimulus is positive or negative, liked or disliked, is arguably the most basic judgment people make regarding any stimulus^10^. As such, it is unsurprising that affective valence influences gaze behavior. Healthy participants show an overall preference for looking at certain types of positive stimuli such as smiling compared to sad faces^11,12^, positively valenced compared to neutral images from the IAPS database^13,14^ and attractive faces^15,16^. This preference typically increases with age, so that older adults show stronger preference for looking at smiling faces and away from angry faces^17–19^. Moreover, healthy participants who score higher on the Life Orientation Test Optimism Scale^20^ look more at neutral skin content and less at melanomas^21^.

Several studies, including meta-analyses^22,23^, that examined gaze preferences of patients with major depressive disorder found that, in comparison to healthy participants, patients look more towards sad faces^24^ and negatively valenced IAPS images^25,26^ and less towards smiling faces and positively valenced images^22,23^.

These findings are often used to argue that while healthy participants have a general preference for looking at positive stimuli, MDD patients have either no preference or a preference for negative stimuli^23^. However, so far, empirical studies of both healthy participants and MDD patients typically considered only one type of positive and negative stimuli in each experiment (see reviews^22,23^). Moreover, when studies included multiple types of positive or negative stimuli such as different emotional facial expressions, or IAPS images engendering sadness or threat^18,27^, the consistency of positivity preference across stimuli categories was not considered analytically. Thus, previous studies did not examine whether preference for looking at more positive stimuli is content dependent (e.g., a specific preference for happy compared to neutral faces) or a general tendency across different visual stimulus types^23^.

To examine if preference for positively valenced stimuli is a general phenomenon, the current study takes an individual differences approach^28,29^. We ask whether, across various types of stimuli, individuals consistently differ in the degree to which stimuli's affective valence influences their gaze preference. If true, this constitutes direct evidence that gaze preference for positive stimuli is, as was previously implied but not directly tested, a general individual tendency^23^. This approach also allows us to examine whether gaze preference for positive stimuli is stable over time, as well as across types of stimuli, qualifying it as a trait. Finally, we complement our individual differences approach with the group differences approach employed in previous studies^24,26^ examining the construct validity of positivity preference by comparing MDD patients' and healthy participants' positivity preference and its modulation by treatment.

We conducted two longitudinal test–retest experiments. In each experiment, gaze preference for more positive stimuli was measured across 7 stimulus categories (happy, angry, fearful or sad emotional faces versus neutral faces, rotten versus appealing food, attractive versus less attractive faces and positive versus neutral images). The first experiment examined in healthy individuals the stability of positive gaze preference between categories and with measurements weeks apart. The second experiment examined the stability of positivity gaze preference across a period of months, whether individuals with MDD exhibit reduced positivity preference, and whether two months of SSRI treatment is accompanied by a change in gaze preference.

To presage, our results indicate that both healthy individuals and individuals with MDD exhibit consistent differences in preferential looking at positive stimuli. These differences are consistent across types of stimuli; individuals showing larger preference for positive stimuli in one category show larger preference for positive stimuli in other categories. Likewise, individual differences are consistent across time, with individuals' level of positivity preference correlating strongly across two experimental sessions taken weeks and months apart. Finally, we show that individuals with MDD exhibit less positivity preference before treatment but their positivity preference significantly increases following treatment. These studies are therefore the first to establish that gaze positivity preference is a trait that is stable over time and reflects the affective state of individuals with MDD.

## Experiment 1

### Participants

Thirty Hebrew University students (23 female, mean age = 22.43, SD = 2.76) participated in the experiment in exchange for 40 NIS (approximately $10) or course credit. The number of participants was the target number stated in the preregistered analysis plan (https://aspredicted.org/HGR_5BN). All participants had normal or corrected to normal vision and provided informed consent prior to participating in the experiment. Given this sample size, a sensitivity analysis indicated 80% statistical power for detecting correlations with $$\rho$$-values of 0.47 or more.

### Stimuli and apparatus

Two hundred and thirty-four images were used as stimuli in the experiment, divided into 117 pairs each belonging to one of seven categories. One image in each pair had a more positive emotional valence than the other. In four categories an emotional facial expression, either angry, fearful, sad or happy, was matched with a neutral facial expression (the angry, fearful, sad and happy images categories respectively) all drawn from the Karolinska Directed Emotional Faces database^30^ (KDEF). There were 10 pairs from each emotional category. The emotional expressions in each pair were displayed by the same actor, with five male and five female actors in each category for a total of 40 unique actors. The fifth category (Attractive faces) included 20 pairs of female faces from the Oslo Face Database^31^. Each pair included a face that was rated as one of the 30 most attractive (mean attractiveness rating 5.49, SD = 0.55) and a face that was rated as one of the 30 least attractive faces (mean attractiveness rating 3.03, SD = 0.59) among the 103 female faces included in this database. The sixth category (IAPS images) included 27 pairs of images drawn from the International Affective Picture System^32^ (IAPS). Each pair included one of the 30 most positively valanced images in the IAPS database (mean valence 8.01, SD = 0.18; e.g., images of puppies, dolphins or a baby. For a full list of IAPS image numbers see Supplementary Table S6) matched with a neutral image (mean valence 5.01, SD = 0.44; e.g., images of mushrooms, a fork or a secretary). The seventh category (Food images) included 30 pairs of images of food items. The thirty images of food items with the highest palatability rating (mean palatability rating 79.73, SD = 3.00) among the food images in the Food-Pics-Extended database^33^ were paired with images of rotten food obtained through Google images.

Participants filled out five self-report questionnaires: the Life Orientation Test-Revised^34^ (LOT-R), the anhedonia subscale from the Computerized Adaptive Test Personality Disorder Static Form^35^ (CAT-PD-SF) , the Attitudes Toward Emotions Scale^36^, the anxiety subscale of the NEO PI-R^37^, and the Social Phobia Inventory^38^ (SPIN).

Stimuli were presented on a 14-inch screen, while gaze position was tracked using SMI 250RED (SansoMotoric Instruments Inc, Teltow, Germany), installed on a DELL laptop.

### Procedure

Each participant provided informed consent prior to beginning the experiment in accordance with the Hebrew University ethics regulations. During the experiment, participants were positioned approximately 60 cm from the monitor. Each participant first performed a calibration and validation sessions of five points (implemented by the experimental software provided by SMI; SansoMotoric Instruments Inc) at the beginning of each session. All the data analyzed here was obtained from recordings with an average absolute global validation error of less than 1 degree of visual angle. Recording sample rate was 250Hz. Analysis was based on fixations parceled from the data by the software provided by SMI (BeGaze, SansoMotoric Instruments Inc). Fixations were detected using peak velocity threshold of 40°/s and minimum duration of 50 ms (default SMI implementation).

Immediately after the calibration and validation procedure, participants viewed the 234 trials of the Image Viewing task, where each of the 117 pairs of stimuli appeared twice, once with the more positive image on the right and once on the left. Trials were presented in random order. Each trial started with a fixation cross displayed at the center of the screen until the participant fixated on it for 300 ms, followed by the images displayed for three seconds, after which there was a 500 ms blank screen until the next trial initiated. Each image spanned approximately 10 by 10—Degrees of Visual Angle (DVA), and images were 6.5 DVA apart, equidistant to the left and right of the screen center. Participants were instructed to first fixate on the fixation cross and then freely view the images.

Participants completed two sessions of the Image Viewing task, conducted one to four weeks apart (M = 12.8 days, SD = 6.10, range 6–28 days). After completing the second session of the Image Viewing task, participants filled out the five self-report questionnaires. Participants were then debriefed and received the reimbursement (payment or course credit). The experiment was approved by the Hebrew University ethics committee.

### Data preparation

According to the preregistered analysis plan, three participants whose eyes could not be tracked during more than 15% of the task duration on at least one of the experimental sessions were excluded from analysis. Likewise, trials in which less than three fixations were recorded (2.43% of trials) were excluded from analysis.

For each trial, preference for the positive image was calculated as the percent of time in which the participant looked at the area of the more positive image minus the percent of time looking at the other image. Preference values across all trials within each condition were then averaged for each participant.

## Results

**Figure 1:**
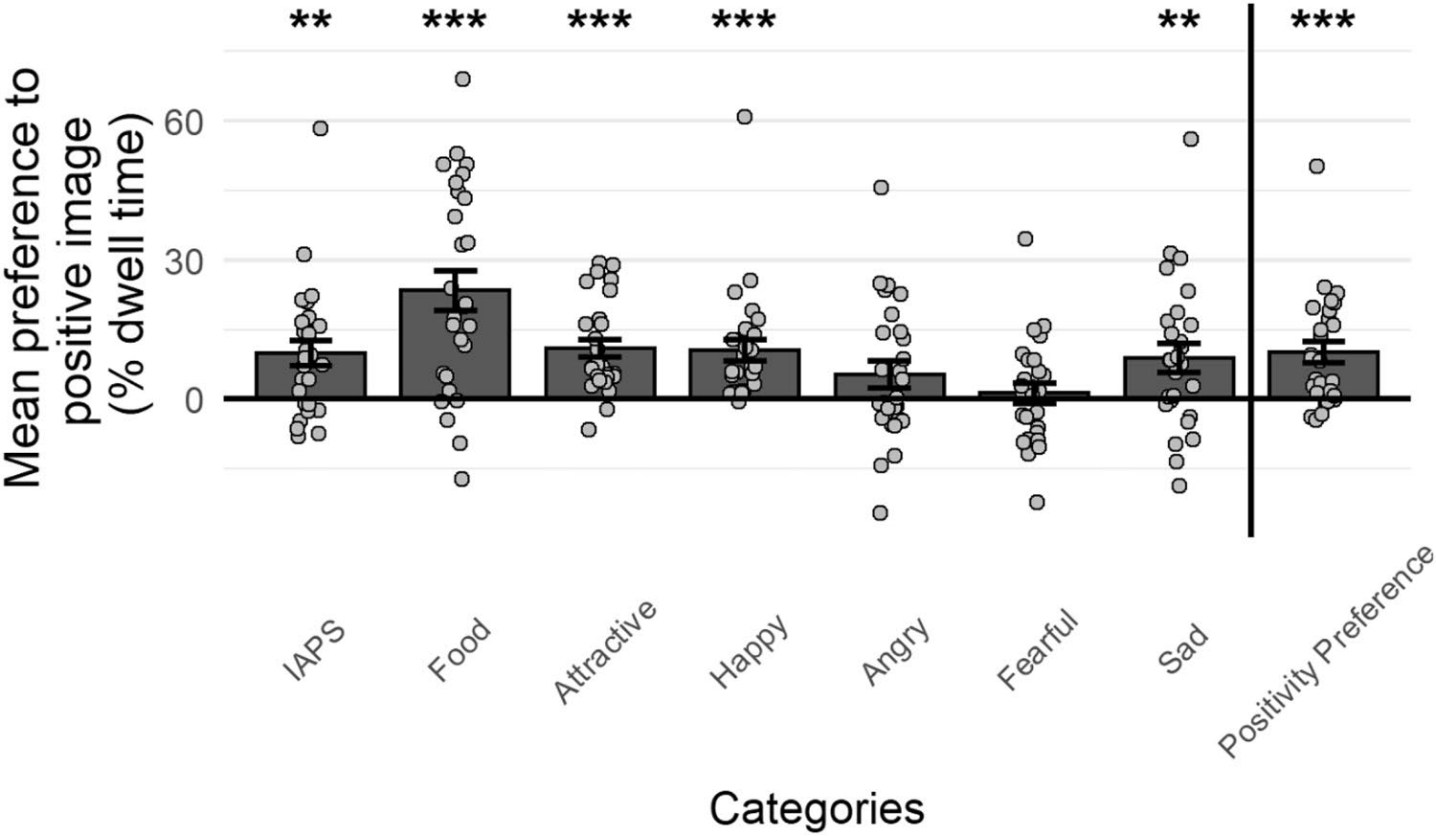
Positivity preference in each category—Mean difference between the percent of fixation time on the positive images and the non-positive images across participants in each category. Each dot reflects a participant’s positive preference value in a specific category. Error bars reflect standard errors across participants. Positivity-Preference, the rightmost category, is the average difference across all categories. ***p < 0.001, **p < 0.01.

As a group, participants looked significantly more at the more positive images in five of the seven categories (mean difference = 8.9–23.36%, all *p* < 0.009; Fig. 1), with no significant difference in the remaining categories (fearful vs. neutral faces and angry vs. neutral faces; Fig. 1). The statistics are detailed in Supplementary Table S1.

**Figure 2:**
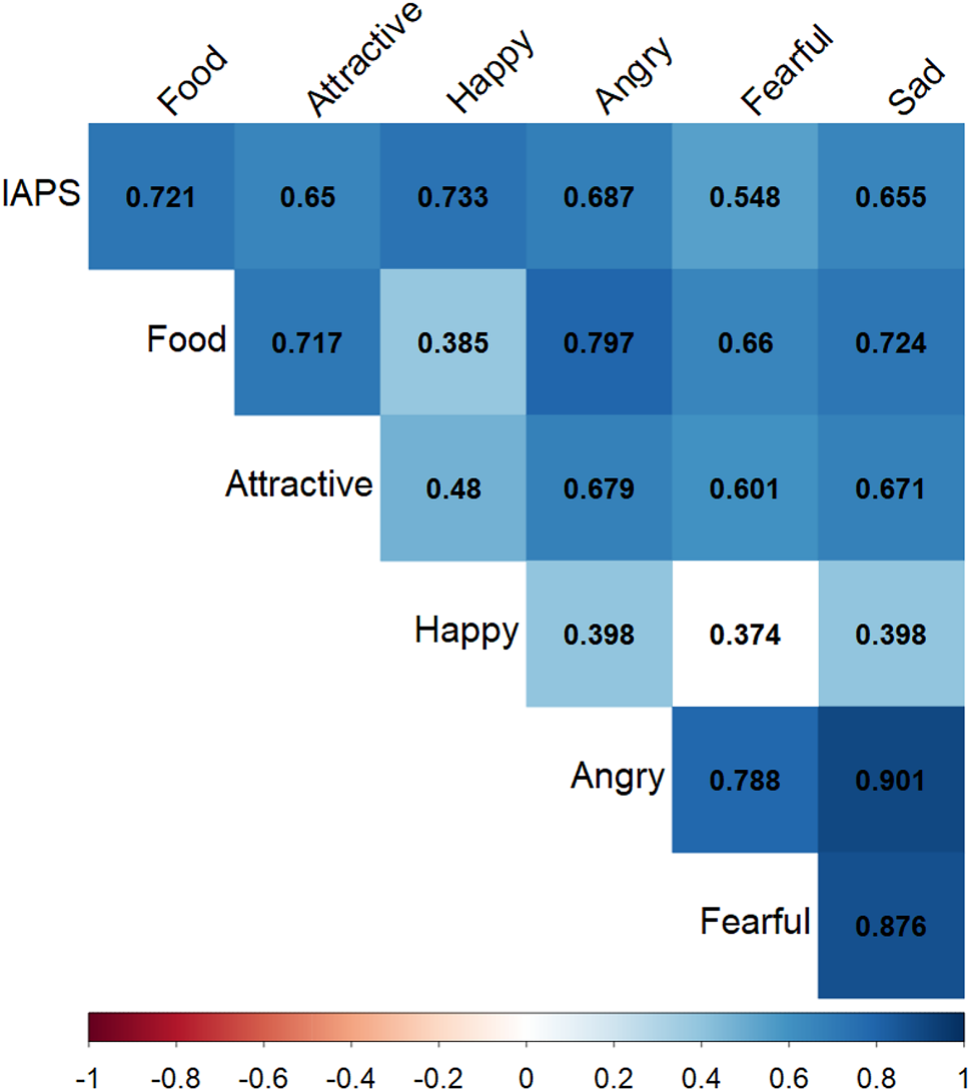
Participants’ preference is correlated across categories—Correlations between participants’ preference values across stimulus categories. Squares’ color reflects the correlation coefficients —darker blue reflects higher coefficients. White squares reflect non-significant correlation.

**Figure 3:**
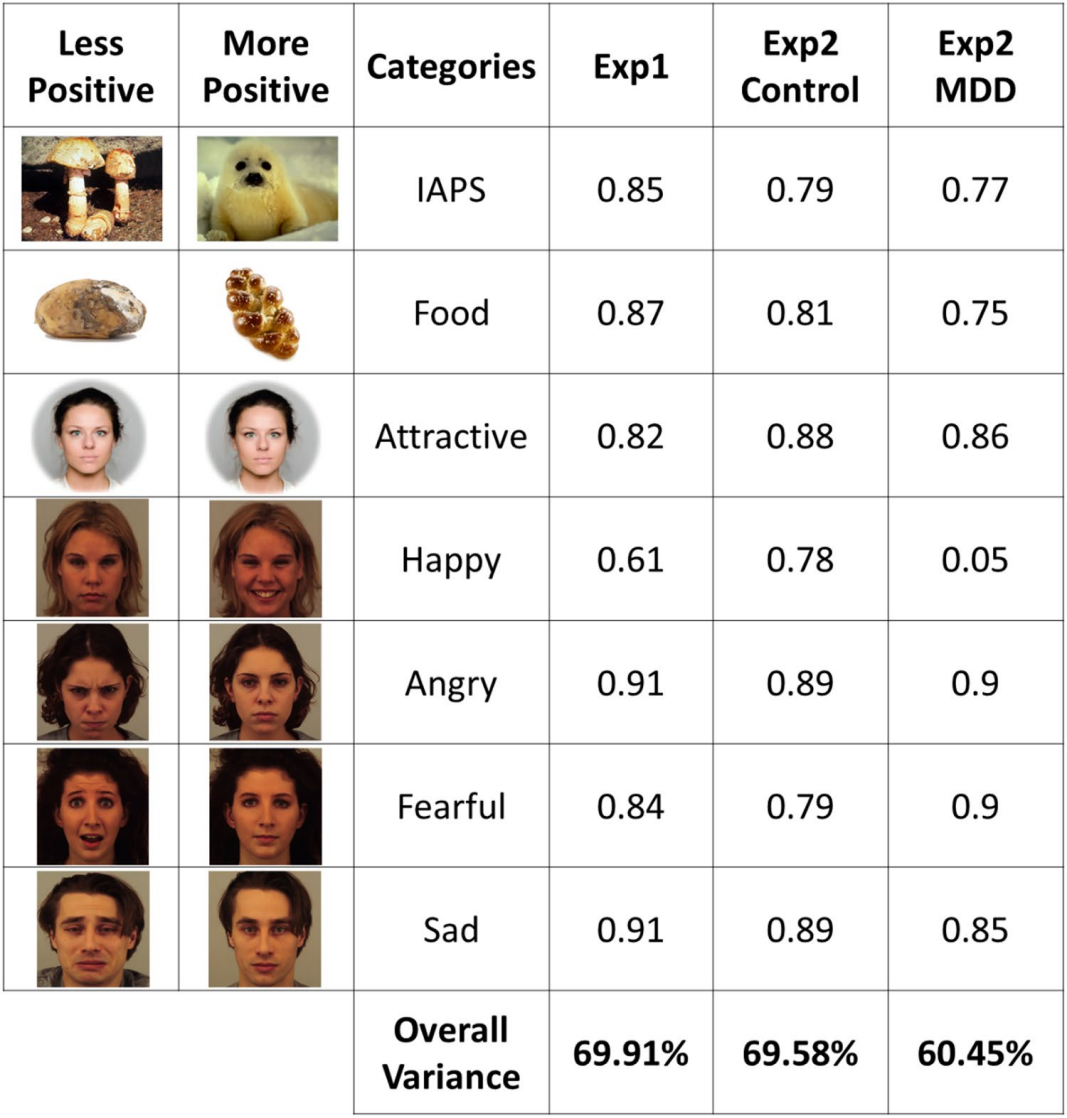
Principal component loadings—Participants’ preferences to the different categories are loaded onto a single component in principal component analysis. The figure presents the loadings of the first PCA component in experiment 1, experiment 2 only for control participants and experiment 2 only for MDD patients. The bottom line shows the proportion of variance explained by the first PCA component. Due to copyright limitations, the example of "attractive faces" includes the same face twice, rather than two different faces (copyright Leknes Affective Brain lab according to the user agreement form). The emotional faces presented are from the KDEF dataset (copyright Karolinska Institutet, Psychology section; images IDs—AF25HAS, AF25NES, AF20ANS, AF20NES, AF13AFS, AF13NES, AM25SAS and AM25NES; https://kdef.se/faq/using-and-publishing-kdef-and-akdef).

Participants’ preference for more positive images was significantly and positively correlated across categories in 20 out of 21 pairwise correlations (*r* between 0.374 and 0.901; in all 21 cases *p* < 0.055; see Fig. 2 and Supplementary Table S2). Further, a principal components analysis indicated that preference for positive images in each category loaded strongly (component loadings between 0.61 and 0.91, see Fig. 3) on a single factor that accounted for 69.91% of the overall variance (see Fig. 3). Factor analysis using a model with one factor revealed a comparative fit index of 0.87. To avoid overfitting to the sample, the general positivity preference scores were computed based on the unweighted average preference for the more positive images across all categories. Notably, our original hypothesis (see preregistration) suggested that the emotional expression categories and motivational categories (IAPS, food and attractiveness) will load onto two factors, however, the correlations and PCA clearly support the existence of only one significant factor.

**Figure 4:**
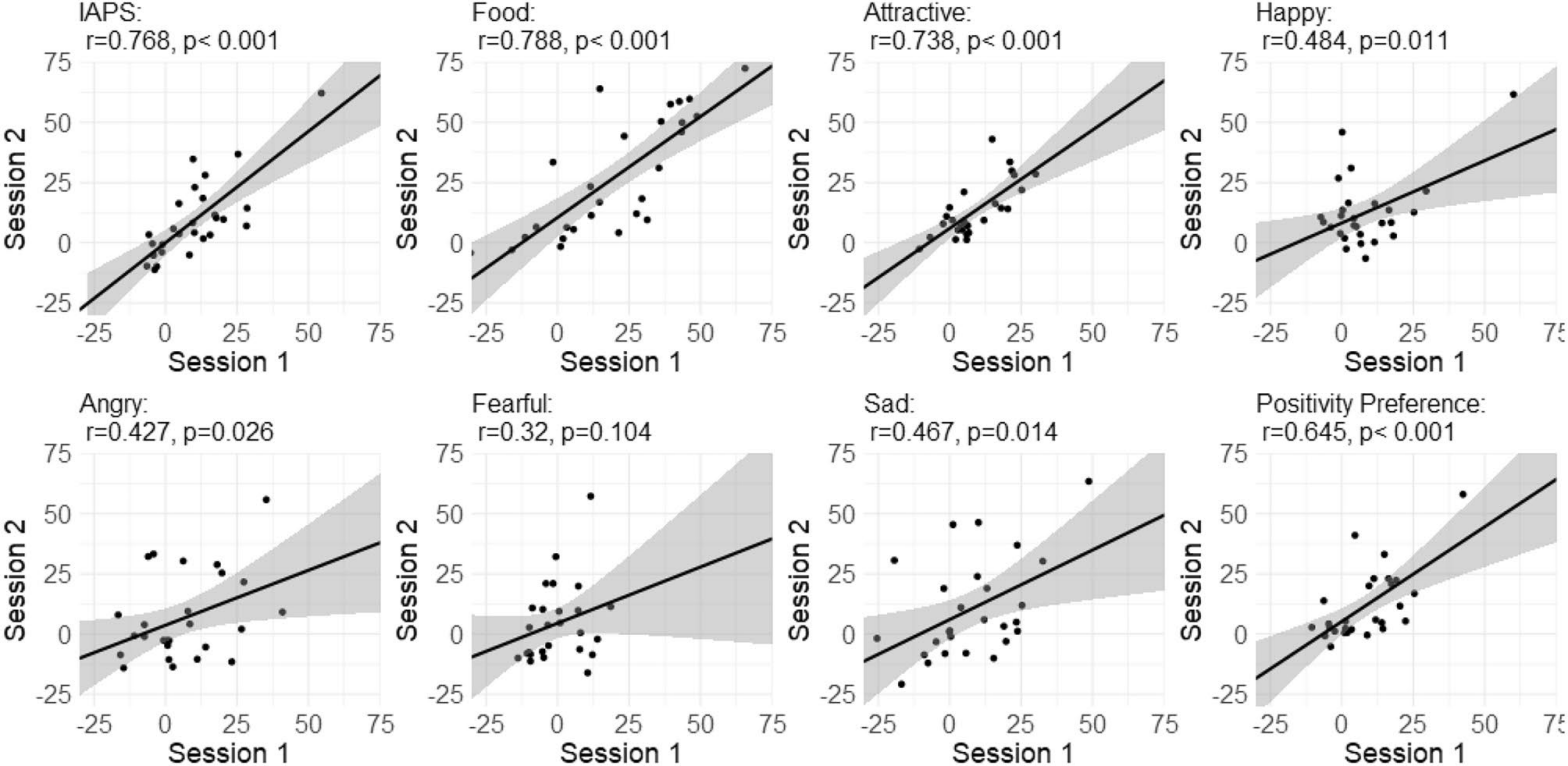
Reliability of positive preference across time in all categories—participant-wise correlations between session 1 and session 2 in each category. Last panel (bottom right) shows the results of the mean positive preference across categories.

Positivity preference scores, the average preference for the more positive images across all categories, in the two sessions were significantly correlated across participants (*r* = 0.645, *p* < 0.001; see Fig. 4). Likewise, preference for single categories was significantly correlated across the two sessions in all categories (0.427 < *r* < 0.788, *p* < 0.027) except fearful faces (*r* = 0.320, *p* = 0.104). The statistics are detailed in Supplementary Table S3.

Individuals’ positivity preference was not significantly related to any of the self-reported scales we examined (all *p* > 0.16; see supplementary Table S4). Likewise, none of the 56 pairwise correlations between self-reported scales and positivity preference in any image category reached statistical significance (*r* between –0.361 and 0.259, all *p* > 0.064).

## Experiment 2

### Participants

The cohort consisted of thirty patients (10 men, 20 women, Mage = 34.8, SD = 10.9) with current MDD diagnosis as established by a senior psychiatrist (RA) based on the DSM-V criteria and 23 healthy participants (12 men, 11 women, Mage = 32.5 SD = 10.2). There was no significant difference in age between the groups (*t*(51) = − 0.513, *p* = 0.61, 95% *CI* [− 7.29, 4.32], *d’* = 0.14) that completed the first session of the experiment. Fourteen MDD patients and 14 healthy participants completed an additional second session. Data collection for this experiment coincided with the advent of the COVID-19 pandemic, leading to an unusually high dropout rate as many participants could not complete the second session within a reasonable timeframe. For session 1, given this sample size, a sensitivity analysis indicated 80% statistical power for detecting a difference between groups with Cohen’s d-values of 0.7 or more. For session 2, a sensitivity analysis indicated 80% statistical power for detecting a difference between groups with Cohen’s d-values of 0.97 or more. Regarding the difference between the two sessions within same participants, a sensitivity analysis indicated 80% statistical power with Cohen’s dz-values of 0.7 or more.

Inclusion criteria for all participants included: 1. Age between 18–65 years old. 2. Participants able to complete, understand and sign written consent forms. 3. Normal or corrected to normal vision. Exclusion criteria for all participants included: 1. History of neurological disorders. 2. Past diagnosis of attention deficits. Inclusion criteria for the MDD participants included: 1. Current DSM-V diagnosis of MDD. 2. Only patients who received zero to two antidepressant medications before enrolment were recruited. All patients were willing to start or to change their current antidepressant medication. Naive patients received sertraline up to 200 mg. Patients who were taking another SSRI were switched to sertraline and patients who were taking sertraline were switched to another SSRI. 3. Eligible for SSRI treatment. Exclusion criteria for MDD participants included additional psychiatric diagnosis from the axis I (Mental Health and Substance Use Disorders). Exclusion criteria for healthy controls included history or current diagnosis of any psychiatric disorder. All participants provided informed consent prior to participating in the experiment. Participants received reimbursement of 150 NIS (approximately $40).

### Stimuli and apparatus

The stimuli presented during the eye-tracking task were identical to those used in Experiment 1. Stimuli were presented on a 24-inch screen, while gaze position was tracked using SMI 250RED (SansoMotoric Instruments Inc, Teltow, Germany), installed on DELL laptop. All participants additionally completed the Hamilton Depression Rating Scale ^39^ during each experimental session.

### Procedure

Each participant provided informed consent prior to beginning the experiment in accordance with the Tel-Hashomer Helsinki committee regulations. Each session of this experiment was embedded in an approximately 4.5 hours long experimental session during which participants provided multiple clinical assessments, performed additional cognitive tasks, and completed EEG recording and MRI scanning sessions. The procedure of the Image Viewing task was identical to Experiment 1, except that each image size was approximately 15 by 15—Degrees Of Visual Angle (DVA), positioned 10 DVAs apart (The variation from the previous experiment was the result of the different screen employed in this experiment).

Participants completed the Image Viewing task twice, in two sessions approximately two months apart (*M* = 71.89 days, *SD* = 32.25, range 40–186 days). MDD patients underwent SSRI antidepressants treatment between the two sessions. The experiment was approved by the Tel-Hashomer Helsinki committee.

### Data preparation

Three participants (two MDD patients and one control) who had less than 75% valid gaze samples in either session were excluded from further analyses according to the preregistered analysis plan (https://aspredicted.org/blind.php?x=UEG_MMJ). Because eye tracking performance is typically noisier in atypical populations, such as MDD patients, we used a lower criterion for valid gaze sample in this experiment, compared to the 85% criterion in experiment 1. In addition, trials in which less than 3 fixations were detected were excluded from analysis (1.03% of trials). As in experiment 1, preference for the positive image was calculated as the difference between the percent of time participants looked at the area of the more positive image minus the percent of time on the other image. The mean preference for positive images was then averaged across all trials from the same category.

### Results

#### The Hamilton Depression Rating Scale

MDD patients scored significantly higher than the healthy participants on the Hamilton Depression Rating Scale both in the first session (MDD patients M = 17.04, SD = 7.37, Control M = 1.41, SD = 1.56, *t*(48) = 9.76, *p* < 0.001, 95% *CI* [12.41, 18.87], *d’* = 2.94) and the second session (MDD patients M = 6.69, SD = 6.03, Control: M = 1.57, SD = 1.95, *t*(25) = 3.01, *p* = 0.006, 95% *CI* [1.62, 8.62], *d’* = 1.14). MDD patients’ Hamilton depression rating scale scores were significantly lower in the second session compared to the first session (*t*(12) = 3.12, *p* = 0.009, 95% *CI* [2.84, 15.93], d’ = 9.39), reflecting a significant improvement in depression severity.

#### Gaze positivity preference

In the first session, healthy control participants looked significantly more at the positive images in six of the seven categories (mean difference = 9.25–26.85%, all *p* < 0.044), with no significant difference in the remaining category (fearful vs. Neutral faces). On the other hand, MDD patients looked in the first session significantly more at the positive images only in two out of the seven categories (appealing vs. Rotten food, mean difference = 13.18%, *p* = 0.006, and attractive vs. unattractive faces, mean difference = 6.78%, *p* = 0.043). In the second session, healthy controls looked significantly more at positive images in four out of the seven categories (mean difference = 13.54–22.57%, all *p* < 0.023), with no significant difference in the remaining three categories (angry vs. Neutral, sad vs. neutral and fearful vs. Neutral faces), while MDD patients looked significantly more at positive images in six out of the seven categories (mean difference = 8.43–29.94%, all* p* < 0.048) with no significant difference in the remaining category (fearful vs. Neutral faces). For full statistics see Supplementary Table S1 and Fig. S1.

**Figure 5:**
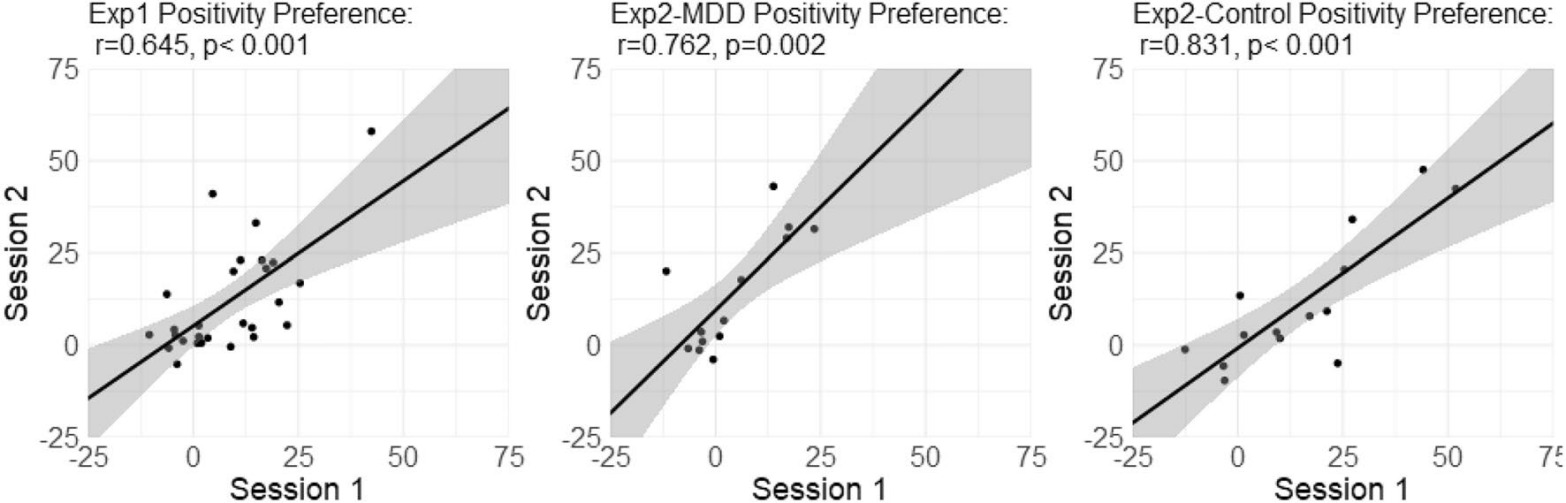
Stability of mean positivity preference across time—correlations between sessions in both experiments for mean positive image preference. In experiment 2 results are presented for control and MDD patients separately.

As in experiment 1, individuals’ positivity preference was correlated across stimulus categories. In the first session, the pairwise correlations between preference in different categories were significantly positive in 19 out of 21 pairwise correlations among the healthy controls and 13 out of 21 among the MDD patients (see Supplementary Table S2). Principal components analysis in healthy control participants indicated that preference for positive images in each category loaded strongly (component loadings between 0.79 and 0.89, see Fig. 3) on a single factor, explaining 69.58% of the variance (factor analysis using a model with one factor revealed a comparative fit index of 0.82). PCA and factor analyses were computed on the data of the first session, due to the low sample size in the second session. Similarly, for the MDD patients, principal components analysis indicated that preference for positive images in six of the seven categories loaded strongly (component loadings between 0.56 and 0.80, see Fig. 3) on a single factor which explained 60.45% of the variance (comparative fit index of 0.77 for a model with one factor). Notably, preference for smiling faces over neutral faces was not loaded on the same factor for MDD patients, with a loading of 0.05. Across time, the mean positive image preference was significantly correlated between the two sessions for both the healthy controls (*r* = 0.831, N = 14, p < 0.001) and the MDD patients (*r* = 0.762, N = 13, *p* = 0.002; see Fig. 5).

**Figure 6:**
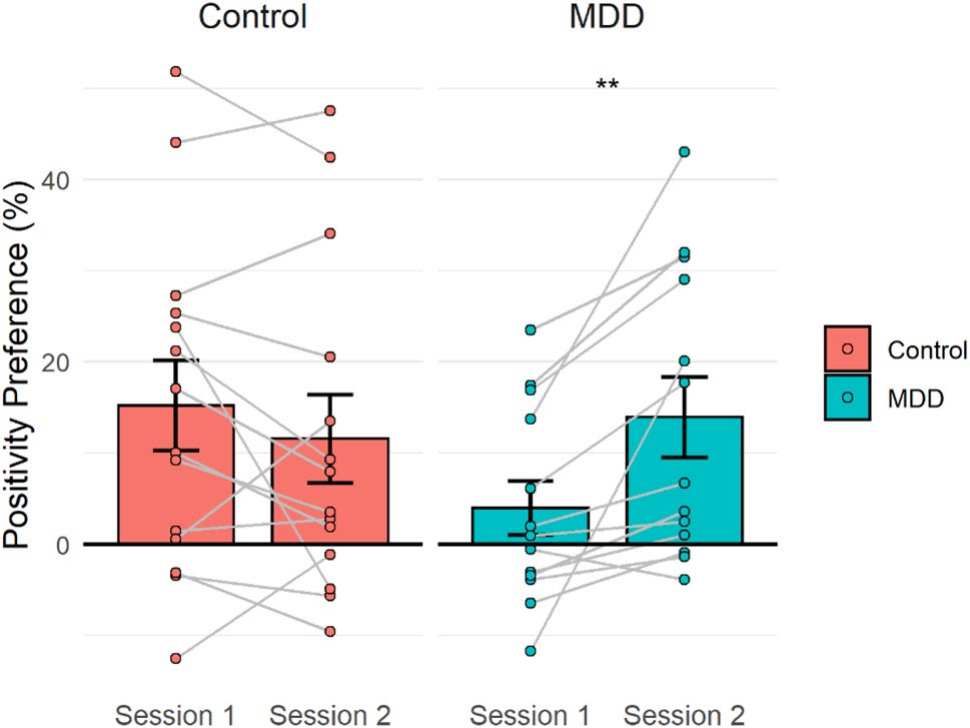
Mean positivity preference across time in control participants and MDD patients—bars represent the averages of each group in each session and error bars represent standard errors. Each pair of dots and the line connecting them represent an individual’s preference in both sessions.

A mixed measures ANOVA on the mean positive image preference revealed a significant interaction between session and group factors (*F*(1,25) = 11.26, *p* = 0.003, ${\eta }_{p}^{2}=0.31$; see Fig. 6), while neither the main effect of group (*F*(1,25) = 0.57, *p* = 0.46, ${\eta }_{p}^{2}=0.02$) or measurement session (*F*(1,25) = 2.47, *p* = 0.13, ${\eta }_{p}^{2}=0.09$) were significant. In the first session, mean positive image preference was significantly lower (*t*(48) = 2.54, *p* = 0.015, 95% *CI* [2.41, 20.85], *d’* = 0.71) for the MDD patients (M = 5.89%, SD = 13.23) compared to the healthy controls (M = 17.52%, SD = 19.17). While in the second session, after patients received treatment, mean positivity preference was not significantly different (*t*(25) = − 0.36, *p* = 0.72, 95% *CI* [− 15.88, 11.18], *d’* = 0.14) between the patients (M = 13.93%, SD = 15.80) and controls (M = 11.58%, SD = 18.14). Patients’ mean positivity preference increased significantly between the two sessions (*t*(12) = 3.48, *p* = 0.005, 95% *CI* [3.73, 16.2], *d’* = 0.97), while there was no significant change for control participants (*t*(13) = − 1.27, *p* = 0.23, 95% *CI* [− 9.77, 2.55,], *d’* = 0.34).

## Discussion

Two experiments exhibited consistent individual differences in gaze positivity preference across types of stimuli (emotional faces, attractive faces, appealing food and positively valenced images) and time (weeks in experiment 1 and months in experiment 2). Furthermore, before treatment, MDD patients showed less positivity preference than control participants. In a subset of MDD patients’ who were available for testing after treatment positivity preference was significantly increased compared to the first session and was no longer significantly different from control participants' positivity preference. Thus, gaze positivity preference can be considered an individual trait influenced by the individual’s mental state.

Previous work on (not gaze related) positivity bias found biases in several domains of cognitive processing, including memory^40^, interpretation^41^ and attention^42,43^. For attention, positivity bias was studied using the dot-probe task and shown to vary between age groups^18^ and patient populations^44^ yet its stability across time and stimuli remained uncertain due to low reliability of the task^45,46^. The current study employed a reliable eye-tracking measure and showed a general positivity bias in gaze deployment, which differs consistently between healthy individuals of similar age and varies with a psychiatric condition (MDD).

Previous findings of differences in gaze preference between individuals with major depressive disorder and healthy participants were primarily based on one or a few stimulus types^22,23^. Studies that included more than one type of stimuli were mainly concerned with different facial expressions of emotion^47^. For example, Doque and Vázquez^47^ displayed emotional and neutral faces and found that individuals with MDD looked more at sad faces and less at happy faces. The current study found that positive gaze preference is a general trait (stable across 7 stimulus types) which is lower in MDD patients compared to healthy participants across various stimulus types. Moreover, after receiving SSRI treatment for two months, MDD patients’ positivity preference increased significantly, reaching levels similar to those of healthy participants. These findings extend a previous study that revealed higher gaze preference for positive images among medicated MDD patients compared to nonmedicated patients^48^. Importantly, the absence of a nonmedicated control group in our experiment raises the possibility that the observed improvement may be attributed to spontaneous recovery, or other factors, rather than a causal effect of SSRIs^49–52^.

Previous literature did not reach consensus as to whether positive and negative preference effects in MDD patients are manifestations of the same underlying construct^23,53^. In other words, it is not clear if preference towards positive stimuli is the same as avoidance of negative stimuli. Here, we show that for healthy individuals, preference towards positive stimuli as measured by the conditions in which a positive stimulus was paired with a neutral one (happy and neutral face, positive and neutral IAPS images) and avoidance of negative stimuli, as measured by conditions in which a negative stimulus was paired with a neutral stimulus (angry, fearful and sad faces paired with neutral faces) loaded strongly on the same general factor. Thus, in healthy individuals, positive gaze preference and negativity avoidance were related and likely stemmed from overlapping underlying constructs. Previous research has also proposed a hypothesis suggesting that depression can be characterized by two distinct factors: motivational and emotional^54^. Building upon this notion, we anticipated observing a distinction between two types of stimuli categories: emotional expressions (e.g., face stimuli) and motivational categories (e.g., IAPS, food, and attractiveness). However, findings from both experiments suggest that gaze preferences are primarily influenced by a single factor.

In MDD patients, positive gaze preference was highly correlated in all categories beside one – happy faces. Previous studies have identified distinct processing patterns for happy faces in individuals with depression, particularly when comparing their visual processing of happy and sad faces^55–57^. For example, one study has revealed that prior to treatment, depressed individuals exhibited higher responses in visual areas when presented with sad stimuli of increasing intensity. However, this heightened response was not observed in response to happy stimuli. Therefore, while positivity preference in most categories   is consistent among MDD patients, the preference for positive facial expressions appears to be less linked to the same underlying construct.

Recent studies found high test–retest reliability of gaze preference towards semantic categories such as faces and text^8,9^, smiling faces^12,27^ and positive natural scenes^27^. These eye-tracking findings, similar to the current findings, stand in contrast to the low test–retest reliability of more traditional dot-probe paradigms (though novel computational methods may improve dot-probe paradigms' reliability^58^) which is the standard measurement paradigm for attentional bias^45,46^. Furthermore, although previous research demonstrated that observers are capable to a certain degree of self-reporting their visual preferences^59^, directly measuring these preferences offers a more reliable and objective measure. Thus, the current study provides a tool for reliable measurement of overt attentional bias for positive stimuli, with potential applications across various populations, including MDD patients and children. Future research should investigate methods to enhance the tool’s sensitivity for specific objectives, such as selecting image categories with strong and reliable positivity preference. For example, the current study suggests that food and attractiveness may lead to more reliable and strong preference. Future studies with larger sample sizes should compare positivity preference in various stimulus types. In experiment 2, the larger recovery in Hamilton scores compared to the gaze positivity preference highlights the potential for improvement in assessing positivity preference. The use of eye tracking could be superior to questionnaires when subjects have language and communication limitations. If positivity preference will be shown to have a causal effect on participants mental conditions (but see^60^) it could potentially be a target for treatment procedures, training participants to focus more on positive images (similarly to attentional bias modification^12^).

Likewise, future studies may investigate whether gaze positivity preference is related to positivity biases in other domains, such as positive interpretation bias^41,61^ or how individuals behave in ambiguous situations^62^. Indeed, the lack of positivity preference in MDD patients reported in the current study, might be related to the tendency of individuals with depression to interpret ambiguous situations more negatively^63^. Notably, the current study did not find correlations with self-reported optimism (failing to conceptually replicate Isaacowitz's study^21^). However, it is possible that because gaze positivity preference is closely related to which visual information one collects moment by moment, gaze positivity preference would more directly influence immediate decisions and interpretations that are based on visual information, rather than one's broader perspective on the world.

The current study has several limitations. First, it used a controlled experimental paradigm and the findings may not generalize to real-life scenarios in which multiple cluttered stimuli are simultaneously present. Future studies should employ more natural conditions, possibly by using wearable eye-tracking devices^64^. Second, although the study examined positivity preference stability across months, preference may change across a lifespan^65^. Third, while we included 7 stimuli categories, much beyond what was done in previous studies, there are many other types of positive stimuli (e.g., money) and negative stimuli (e.g., threatening images) which were not included. Forth, the sample sizes in both experiments are modest, potentially limiting the precision of effect size estimates. However, the consistent findings across two experiments, and the overall high correlations among gaze preference measures indicate robust trends, supporting the claim about a stable and general gaze positivity preference. Nevertheless, the results of Experiment 2, concerning the alteration in gaze positivity preference among individuals with MDD, should be treated with caution due to the modest sample size. Finally, our experimental design treated the positive or negative valence of each stimulus as a constant, while in practice the affective values of stimuli likely differ between individuals. In the current study, such differences increase measurement error, but future work could achieve more precise measurement by including individual ratings of affective value as additional predictors.

In conclusion, the current study shows that preferential looking at more positive stimuli is an individual trait, consistent across different stimulus types and stable across time. Moreover, positive preference is lower in patients with major depressive disorder compared to healthy participants and returns to normal levels after treatment. Thus, gaze positivity preference can be viewed as a trait that determines the amount of positive visual information that is selected for processing and therefore could modulate the observer's emotional state. Indeed, positivity preference differs in abnormal affective states and responds to treatment.

### Supplementary Information

Supplementary Information.

## Data Availability

Both experiments were preregistered. Deidentified data for both experiments are accessible at https://osf.io/mkh8g/?view_only=2e6777cd2a3340aea088b550831a8f2b. Requests for the analysis scripts and stimuli can be sent to the corresponding author.

## References

[1] Osterberg GA (1935). Topography of the layer of the rods and cones in the human retina. Acta Ophthalmol..

[2] *Diagnostic and Statistical Manual of Mental Disorders: DSM-5*. (American Psychiatric Association, Washington, D.C, 2013).

[3] Buswell, G. T. How people look at pictures: A study of the psychology and perception in art. (1935).

[4] Yarbus, A. L. *Eye Movements during Perception of Complex Objects*. (Springer, 1967).

[5] Castelhano MS, Mack ML, Henderson JM (2009). Viewing task influences eye movement control during active scene perception. J. Vision.

[6] Henderson JM, Luke SG (2014). Stable individual differences in saccadic eye movements during reading, pseudoreading, scene viewing, and scene search. J. Exp. Psychol. Human Perception Performance.

[7] Luke SG, Darowski ES, Gale SD (2018). Predicting eye-movement characteristics across multiple tasks from working memory and executive control. Mem. Cogn..

[8] Guy N (2019). A novel perceptual trait: gaze predilection for faces during visual exploration. Sci. Rep..

[9] de Haas, B., Iakovidis, A. L., Schwarzkopf, D. S. & Gegenfurtner, K. R. Individual differences in visual salience vary along semantic dimensions. *PNAS* 201820553 (2019). 10.1073/pnas.1820553116. 10.1073/pnas.1820553116 PMC6576124 31138705

[10] Zajonc RB (1980). Feeling and thinking: Preferences need no inferences. Am. Psychol..

[11] Bradley BP (1997). Attentional biases for emotional faces. Cognit. Emot..

[12] Lazarov A, Ben-Zion Z, Shamai D, Pine DS, Bar-Haim Y (2018). Free viewing of sad and happy faces in depression: A potential target for attention bias modification. J. Affect. Disorders.

[13] Pool E, Brosch T, Delplanque S, Sander D (2015). Attentional bias for positive emotional stimuli: A meta-analytic investigation. Psychol. Bull..

[14] Raila H, Scholl BJ, Gruber J (2015). Seeing the world through rose-colored glasses: People who are happy and satisfied with life preferentially attend to positive stimuli. Emotion.

[15] Leder H, Tinio PPL, Fuchs IM, Bohrn I (2010). When attractiveness demands longer looks: The effects of situation and gender. Quart. J. Exp. Psychol..

[16] Leder, H., Mitrovic, A. & Goller, J. How beauty determines gaze! Facial attractiveness and gaze duration in images of real world scenes. *i-Perception* **7**, 2041669516664355 (2016). 10.1177/2041669516664355 PMC5030765 27698984

[17] Nikitin J, Freund AM (2011). Age and motivation predict gaze behavior for facial expressions. Psychol. Aging.

[18] Isaacowitz DM, Wadlinger HA, Goren D, Wilson HR (2006). Selective preference in visual fixation away from negative images in old age? An eye-tracking study. Psychol. Aging.

[19] Isaacowitz DM, Wadlinger HA, Goren D, Wilson HR (2006). Is there an age-related positivity effect in visual attention? A comparison of two methodologies. Emotion.

[20] Scheier MF, Carver CS (1985). Optimism, coping, and health: Assessment and implications of generalized outcome expectancies. Health Psychol..

[21] Isaacowitz DM (2005). The gaze of the optimist. Pers. Soc. Psychol. Bull..

[22] Armstrong T, Olatunji BO (2012). Eye tracking of attention in the affective disorders: A meta-analytic review and synthesis. Clin. Psychol. Rev..

[23] Suslow T, Hußlack A, Kersting A, Bodenschatz CM (2020). Attentional biases to emotional information in clinical depression: A systematic and meta-analytic review of eye tracking findings. J. Affective Disorders.

[24] Gotlib IH, Krasnoperova E, Yue DN, Joormann J (2004). Attentional biases for negative interpersonal stimuli in clinical depression. J. Abnormal Psychol..

[25] Eizenman M (2003). A naturalistic visual scanning approach to assess selective attention in major depressive disorder. Psychiatry Res..

[26] Kellough JL, Beevers CG, Ellis AJ, Wells TT (2008). Time course of selective attention in clinically depressed young adults: An eye tracking study. Behav. Res. Therapy.

[27] Sears C, Quigley L, Fernandez A, Newman K, Dobson K (2019). The reliability of attentional biases for emotional images measured using a free-viewing eye-tracking paradigm. Behav. Res..

[28] Vogel EK, Awh E (2008). How to exploit diversity for scientific gain: Using individual differences to constrain cognitive theory. Curr. Dir. Psychol. Sci..

[29] Bolger N, Zee KS, Rossignac-Milon M, Hassin RR (2019). Causal processes in psychology are heterogeneous. J. Exp. Psychol. General.

[30] Lundqvist, D., Flykt, A. & Öhman, A. Karolinska directed emotional faces. *Cognition Emotion* (1998).

[31] Chelnokova O (2014). Rewards of beauty: The opioid system mediates social motivation in humans. Mol. Psychiatry.

[32] Lang, P. J. International affective picture system (IAPS): Affective ratings of pictures and instruction manual. *Technical report* (2005).

[33] Blechert J, Lender A, Polk S, Busch NA, Ohla K (2019). Food-pics_extended—An image database for experimental research on eating and appetite: additional images, normative ratings and an updated review. Front. Psychol..

[34] Carver, C. S. Life orientation test-revised (LOT-R). *Measurement Instrument Database for the Social Science* (2013).

[35] Simms LJ (2011). Computerized adaptive assessment of personality disorder: Introducing the CAT–PD project. J. Personality Assessment.

[36] Harmon-Jones E, Harmon-Jones C, Amodio DM, Gable PA (2011). Attitudes toward emotions. J. Personality Social Psychol..

[37] Costa, P. T. & McCrae, R. R. *Neo Personality Inventory-Revised (NEO PI-R)*. (Psychological Assessment Resources Odessa, FL, 1992).

[38] Connor KM (2000). Psychometric properties of the Social Phobia Inventory (SPIN): New self-rating scale. Br. J. Psychiatry.

[39] Hamilton M (1960). A rating scale for depression. J. Neurol. Neurosurg. Psychiatry.

[40] Walker WR, Skowronski JJ, Thompson CP (2003). Life is pleasant—And memory helps to keep it that way!. Rev. General Psychol..

[41] Hirsch CR, Mathews A (2000). Impaired positive inferential bias in social phobia. J. Abnormal Psychol..

[42] Brosch T, Sander D, Pourtois G, Scherer KR (2008). Beyond fear: Rapid spatial orienting toward positive emotional stimuli. Psychol. Sci..

[43] Wirth BE, Wentura D (2020). It occurs after all: Attentional bias towards happy faces in the dot-probe task. Atten. Percept. Psychophys..

[44] Peckham AD, McHugh RK, Otto MW (2010). A meta-analysis of the magnitude of biased attention in depression. Depression Anxiety.

[45] Schmukle SC (2005). Unreliability of the dot probe task. Eur. J. Pers..

[46] Chapman A, Devue C, Grimshaw GM (2019). Fleeting reliability in the dot-probe task. Psychol. Res..

[47] Duque A, Vázquez C (2015). Double attention bias for positive and negative emotional faces in clinical depression: Evidence from an eye-tracking study. J. Behav. Therapy Exp. Psychiatry.

[48] Wells TT, Clerkin EM, Ellis AJ, Beevers CG (2014). Effect of antidepressant medication use on emotional information processing in major depression. AJP.

[49] Hegel MT, Oxman TE, Hull JG, Swain K, Swick H (2006). Watchful waiting for minor depression in primary care: Remission rates and predictors of improvement. General Hospital Psychiatry.

[50] Jeuring HW, Huisman M, Comijs HC, Stek ML, Beekman ATF (2016). The long-term outcome of subthreshold depression in later life. Psychol. Med..

[51] Mei G, Li Y, Chen S, Cen M, Bao M (2020). Lower recognition thresholds for sad facial expressions in subthreshold depression: A longitudinal study. Psychiatry Res..

[52] Qiu S, Luo X, Luo Y, Wei D, Mei G (2023). State-dependent alterations of implicit emotional dominance during binocular rivalry in subthreshold depression. PsyCh. J..

[53] Vanderlind WM, Millgram Y, Baskin-Sommers AR, Clark MS, Joormann J (2020). Understanding positive emotion deficits in depression: From emotion preferences to emotion regulation. Clin. Psychol. Rev..

[54] Drysdale AT (2017). Resting-state connectivity biomarkers define neurophysiological subtypes of depression. Nat. Med..

[55] Surguladze S (2005). A differential pattern of neural response toward sad versus happy facial expressions in major depressive disorder. Biol. Psychiatry.

[56] Keedwell PA, Andrew C, Williams SCR, Brammer MJ, Phillips ML (2005). A double dissociation of ventromedial prefrontal cortical responses to sad and happy stimuli in depressed and healthy individuals. Biol. Psychiatry.

[57] Keedwell P (2009). Neural markers of symptomatic improvement during antidepressant therapy in severe depression: Subgenual cingulate and visual cortical responses to sad, but not happy, facial stimuli are correlated with changes in symptom score. J. Psychopharmacol..

[58] Price RB, Brown V, Siegle GJ (2019). Computational modeling applied to the dot-probe task yields improved reliability and mechanistic insights. Biol. Psychiatry.

[59] Guy N, Kardosh R, Sklar AY, Lancry-Dayan OC, Pertzov Y (2023). Do we know our visual preferences?. J. Vision.

[60] Hertz-Palmor N, Yosef Y, Hallel H, Bernat I, Lazarov A (2024). Exploring the ‘mood congruency’ hypothesis of attention allocation—An eye-tracking study. J. Affect. Disorders.

[61] Hirsch C, Mathews A (1997). Interpretative inferences when reading about emotional events. Behav. Res. Therapy.

[62] Rocklage, M. D., Pietri, E. S. & Fazio, R. H. The weighting of positive vs. negative valence and its impact on the formation of social relationships. *J. Exp. Social Psychol. 73*, 65–75 (2017).

[63] Berna C, Lang TJ, Goodwin GM, Holmes EA (2011). Developing a measure of interpretation bias for depressed mood: An ambiguous scenarios test. Pers. Individ. Dif..

[64] Peterson MF, Lin J, Zaun I, Kanwisher N (2016). Individual differences in face-looking behavior generalize from the lab to the world. J. Vision.

[65] Murphy NA, Isaacowitz DM (2008). Preferences for emotional information in older and younger adults: A meta-analysis of memory and attention tasks. Psychol. Aging.

