# Supplementary material for: Individuals vary in their overt attention preference for positive images consistently across time and stimulus types
Source: Sci Rep. 2024 Apr 15;14:8712. doi: 10.1038/s41598-024-58987-8 (PMC11018868; doi:10.1038/s41598-024-58987-8)
Supplement: Supplementary file 1 — Supplementary Information. [file 41598_2024_58987_MOESM1_ESM.docx]

**Individuals vary in their overt attention preference for positive images consistently across time and stimulus types – Supplementary Material**

***Preference for more positive images in different categories***

|  | **Experiment 1** | **Experiment 2** | | | |
| --- | --- | --- | --- | --- | --- |
| **Category** | **Average across sessions** | **MDD – Session 1**  **N=28** | **MDD –**  **Session 2**  **N=13** | **Control – Session 1**  **N=22** | **Control –**  **Session 2**  **N=14** |
| IAPS | **M=9.90%,**  **SD=14.16, t(26)=3.63, p=0.001,**  **95% CI [4.3,15.5],**  **d’=0.7** | M=4.16%,  SD=13.18, t(27)=1.67, p=0.106**,**  95% CI [-0.95, 9.27],  d’=0.32 | **M=11.24%,**  **SD=17.27, t(12)=2.35,**  **p = 0.037, ,**  **95% CI [0.81, 21.68],**  **d’=0.65** | **M=19.34%,**  **SD=17.68, t(21)=5.13,**  **p<0.001,**  **95% CI [11.5, 27.17],**  **d’=1.09** | **M=13.54%,**  **SD=15.7, t(13)=3.23,**  **p = 0.007,**  **95% CI [4.48, 22.6],**  **d’=0.86** |
| Food | **M=23.36%, SD=22.48, t(26)=5.4,**  **p<0.001,**  **95% CI [14.47,32.25],**  **d’=1.04** | **M=13.89%,**  **SD=24.89, t(27)=2.95,**  **p=0.006,**  **95% CI [4.24, 23.54],**  **d’=0.56** | **M=29.94%,**  **SD=26.34, t(12)=4.1,**  **p = 0.001,**  **95% CI [14.03, 45.85],**  **d’=1.14** | **M=26.85%,**  **SD=29.6, t(21)=4.25,**  **p<0.001,**  **95% CI [13.72, 39.97],**  **d’=0.91** | **M=23.57%,**  **SD=34.33, t(13)=2.57,**  **p = 0.023,**  **95% CI [3.75, 43.39],**  **d’=0.69** |
| Attractive | **M=10.93%, SD=10.04, t(26)=5.65, p<0.001,**  **95% CI [6.96, 14.9],**  **d’=1.09** | **M=6.78%,**  **SD=16.9, t(27)=2.12,**  **p=0.043,**  **95% CI [0.23, 13.33],**  **d’=0.4** | **M=8.43%,**  **SD=11.69, t(12)=2.6,**  **p = 0.023,**  **95% CI [1.36, 15.5],**  **d’=0.72** | **M=17.05%,**  **SD=17.43, t(21)=4.59,**  **p<0.001,**  **95% CI [6.96, 14.9],**  **d’=0.98** | **M=13.81%,**  **SD=15.56, t(13)=3.32,**  **p = 0.006,**  **95% CI [4.83, 22.79],**  **d’=0.89** |
| Happy | **M=10.52%, SD=12.25, t(26)=4.46, p<0.001,**  **95% CI [5.97, 15.36],**  **d’=0.86** | M=4.81%,  SD=13.3, t(27)=1.91,  p=0.07**,**  95% CI [-0.35, 9.96],  d’=0.36 | **M=12.78%,**  **SD=20.79, t(12)=2.22,**  **p = 0.047,**  **95% CI [0.22, 25.34],**  **d’=0.61** | **M=24.59%,**  **SD=20.5, t(21)=5.63,**  **p<0.001,**  **95% CI [15.5, 33.67],**  **d’=1.2** | **M=22.43%,**  **SD=19.16, t(13)=4.38,**  **p = 0.001,**  **95% CI [11.37, 33.67],**  **d’=1.17** |
| Angry | M=5.34%,  SD=14.91, t(26)=1.86, p=0.074,  95% CI [-0.56, 11.23],  d’=0.36 | M=3.81,  SD=16.56,t(27)=1.22,  p=0.23,  95% CI [-2.62, 10.23],  d’=0.23 | **M=12.48%,**  **SD=17.07, t(12)=2.64,**  **p = 0.022,**  **95% CI [2.17, 22.8],**  **d’=0.73** | **M=11.95%,**  **SD=26.02, t(21)=2.15,**  **p=0.043,**  **95% CI [0.41, 23.49],**  **d’=0.46** | M=3.1%,  SD=24.89, t(13)=0.47,  p = 0.649**,**  95% CI [-11.27, 17.47],  d’=0.12 |
| Fearful | M=1.27%,  SD=11.19, t(26)=0.59, p=0.561,  95% CI [-.16, 5.69],  d’=0.11 | M=3.93,  SD=17.45, t(27)=1.19,  p=0.24,  95% CI [-2.84, 10.7],  d’=0.23 | M=8.87%,  SD=23.34, t(12)=1.37,  p = 0.196,  95% CI [-5.23, 22.97],  d’=0.38 | M=9.25%,  SD=24.43, t(21)=1.78,  P=0.089**,**  95% CI [-1.53, 20.03],  d’=0.38 | M=1.15%,  SD=25.27, t(13)=0.17,  p = 0.867**,**  95% CI [-13.44, 15.76],  d’=0.05 |
| Sad | **M=8.90%,**  **SD=16.04, t(26)=2.88, p=0.008,**  **95% CI [2.55, 15.24],**  **d’=0.55** | M=3.87%,  SD=20.05, t(27)=1.02,  p=0.316,  95% CI [-3.9, 11.65],  d’=0.19 | **M=13.76%,**  **SD=16.1, t(12)=3.08,**  **p = 0.01,**  **95% CI [4.03, 23.49],**  **d’=0.85** | **M=13.64%,**  **SD=25.23, t(21)=2.54,**  **p=0.019,**  **95% CI [2.45, 24.83],**  **d’=0.54** | M=3.47%,  SD=22.96, t(13)=0.57,  p = 0.582**,**  95% CI [-9.79, 16.73],  d’=0.15 |
| Positive-Preference | **M=10.03%,**  **SD=12.15, t(26)=4.29, p<0.001,**  **95% CI [5.22, 14.83],**  **d’=0.83** | **M=5.89%,**  **SD=13.23, t(27)=2.36,**  **p=0.026,**  **95% CI [0.76, 11.02],**  **d’=0.44** | **M=13.93%,**  **SD=15.8, t(12)=3.18,**  **p = 0.008,**  **95% CI [4.38, 23.48],**  **d’=0.88** | **M=17.52%,**  **SD=19.17, t(21)=4.29,**  **p<0.001,**  **95% CI [9.02, 26.02],**  **d’=0.91** | **M=11.58%,**  **SD=18.14, t(13)=2.39,**  **p = 0.033,**  **95% CI [1.11, 22.06],**  **d’=0.64** |

*Table S1:* ***Preference for more positive images in different categories*** *– Each cell presents the results of a one sample t-test comparing between the positivity preference and zero. Significant results are in bold.*

***Pairwise correlations between preference for more positive images in different categories***

|  |  | **Experiment 1** | **Experiment 2** | | | |
| --- | --- | --- | --- | --- | --- | --- |
| **Category1** | **Category2** | **N = 27** | **MDD –**  **Session 1**  **N = 28** | **MDD –**  **Session 2**  **N = 13** | **Control – Session 1**  **N = 22** | **Control – Session 2**  **N = 14** |
| IAPS | Food | **r=0.721,**  **p < 0.001** | **r = 0.515,**  **p = 0.005** | **r = 0.826,**  **p = 0.001** | **r = 0.638,**  **p = 0.001** | **r = 0.606,**  **p = 0.022** |
|  | Attractive | **r=0.65,**  **p < 0.001** | **r = 0.587,**  **p = 0.001** | **r = 0.628,**  **p = 0.021** | **r = 0.643,**  **p = 0.001** | r = 0.22,  p = 0.451 |
|  | Happy | **r=0.733,**  **p < 0.001** | **r = 0.518,**  **p = 0.005** | r = 0.355,  p = 0.233 | **r = 0.65,**  **p = 0.001** | **r = 0.609,**  **p = 0.021** |
|  | Angry | **r=0.687,**  **p < 0.001** | **r = 0.635,**  **p < 0.001** | **r = 0.74,**  **p = 0.004** | **r = 0.586,**  **p = 0.004** | r = 0.246,  p = 0.397 |
|  | Fearful | **r=0.548,**  **p = 0.003** | **r = 0.549,**  **p = 0.002** | **r = 0.832,**  **p < 0.001** | **r = 0.531,**  **p = 0.011** | r = 0.262,  p = 0.365 |
|  | Sad | **r=0.655,**  **p < 0.001** | **r = 0.593,**  **p = 0.001** | **r = 0.663,**  **p = 0.013** | **r = 0.608,**  **p = 0.003** | r = 0.19,  p = 0.516 |
| Food | Attractive | **r=0.717,**  **p < 0.001** | **r = 0.694,**  **p < 0.001** | r = 0.404,  p = 0.171 | **r = 0.765,**  **p < 0.001** | **r = 0.71,**  **p = 0.004** |
|  | Happy | **r=0.385,**  **p = 0.047** | r = 0.065,  p = 0.743 | r = 0.415,  p = 0.159 | **r = 0.753,**  **p < 0.001** | **r = 0.86,**  **p < 0.001** |
|  | Angry | **r=0.797,**  **p < 0.001** | **r = 0.538,**  **p = 0.003** | **r = 0.812,**  **p = 0.001** | **r = 0.569,**  **p = 0.006** | **r = 0.595,**  **p = 0.025** |
|  | Fearful | **r=0.66,**  **p < 0.001** | **r = 0.613,**  **p = 0.001** | **r = 0.924,**  **p < 0.001** | r = 0.397,  p = 0.067 | r = 0.41,  p = 0.145 |
|  | Sad | **r=0.724,**  **p < 0.001** | **r = 0.469,**  **p = 0.012** | **r = 0.723,**  **p = 0.005** | **r = 0.596,**  **p = 0.003** | r = 0.488,  p = 0.077 |
| Attractive | Happy | **r=0.48,**  **p = 0.01** | r = -0.02,  p = 0.919 | r = 0.008,  p = 0.979 | **r = 0.726,**  **p < 0.001** | **r = 0.65,**  **p = 0.012** |
|  | Angry | **r=0.679,**  **p < 0.001** | **r = 0.653,**  **p < 0.001** | r = 0.512,  p = 0.073 | **r = 0.692,**  **p < 0.001** | **r = 0.66,**  **p = 0.01** |
|  | Fearful | **r=0.601,**  **p < 0.001** | **r = 0.79,**  **p < 0.001** | **r = 0.583,**  **p = 0.036** | **r = 0.547,**  **p = 0.008** | r = 0.512,  p = 0.061 |
|  | Sad | **r=0.671,**  **p < 0.001** | **r = 0.602,**  **p = 0.001** | r = 0.44,  p = 0.133 | **r = 0.732,**  **p < 0.001** | **r = 0.607,**  **p = 0.021** |
| Happy | Angry | **r=0.398,**  **p = 0.04** | r = -0.068,  p = 0.729 | r = 0.504,  p = 0.079 | **r = 0.643,**  **p = 0.001** | **r = 0.559,**  **p = 0.038** |
|  | Fearful | r=0.374,  p = 0.054 | r = -0.209,  p = 0.287 | r = 0.384,  p = 0.195 | r = 0.419,  p = 0.052 | r = 0.457,  p = 0.1 |
|  | Sad | **r=0.398,**  **p = 0.04** | r = -0.007,  p = 0.972 | **r = 0.604,**  **p = 0.029** | **r = 0.512,**  **p = 0.015** | r = 0.372,  p = 0.19 |
| Angry | Fearful | **r=0.788,**  **p < 0.001** | **r = 0.802,**  **p < 0.001** | **r = 0.913,**  **p < 0.001** | **r = 0.894,**  **p < 0.001** | **r = 0.898,**  **p < 0.001** |
|  | Sad | **r=0.901,**  **p < 0.001** | **r = 0.852,**  **p < 0.001** | **r = 0.886,**  **p < 0.001** | **r = 0.885,**  **p < 0.001** | **r = 0.926,**  **p < 0.001** |
| Fearful | Sad | **r=0.876,**  **p < 0.001** | **r = 0.739,**  **p < 0.001** | **r = 0.82,**  **p = 0.001** | **r = 0.809,**  **p < 0.001** | **r = 0.887,**  **p < 0.001** |

*Table S2:* ***Pairwise correlations between individuals’ preference for more positive images in different categories*** *– Each cell presents the statistics of a subject-wise correlation between individuals’ positivity preference across two stimulus categories (described in the two left columns). Significant correlations are in bold.*

***Stability of preference for more positive images in different categories across sessions***

| **Category** | **Experiment 1**  **N=27** | **Experiment 2 – MDD**  **N=13** | **Experiment 2 – Control**  **N=14** |
| --- | --- | --- | --- |
| IAPS | **r = 0.768,**  **p < 0.001** | r = 0.45,  p = 0.123 | **r = 0.72,**  **p = 0.004** |
| Food | **r = 0.788,**  **p < 0.001** | r = 0.547,  p = 0.053 | **r = 0.881,**  **p < 0.001** |
| Attractive | **r = 0.738,**  **p < 0.001** | r = 0.176,  p = 0.565 | **r = 0.768,**  **p = 0.001** |
| Happy | **r = 0.484,**  **p = 0.011** | r = 0.473,  p = 0.102 | **r = 0.876,**  **p < 0.001** |
| Angry | **r = 0.427,**  **p = 0.026** | r = 0.461,  p = 0.113 | **r = 0.718,**  **p = 0.004** |
| Fearful | r = 0.32,  p = 0.104 | **r = 0.79,**  **p = 0.001** | **r = 0.736,**  **p = 0.003** |
| Sad | **r = 0.467,**  **p = 0.014** | r = 0.304,  p = 0.313 | **r = 0.728,**  **p = 0.003** |
| Positive-Preference | **r = 0.645,**  **p < 0.001** | **r=0.762,**  **p=0.002** | **r=0.831,**  **p<0.001** |

*Table S3:* ***Pairwise correlations across sessions*** *– Each cell presents the correlation coefficient and p value of the correlation between the two testing sessions in a certain stimulus category. Significant results are in bold.*

***
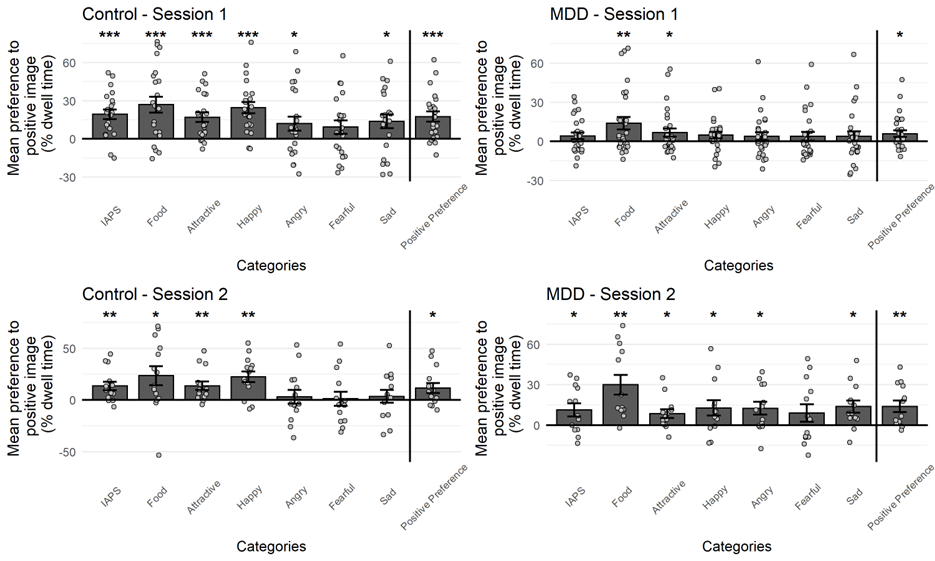
Preference for more positive images in different categories – Experiment 2***

*Figure S1: Average* ***positivity preference in each stimulus category in each session –****Each dot reflects a participant’s positive preference value in a specific category. Error bars reflect standard errors across participants. Positive-Preference, the rightmost category, is the average difference across all categories. *** denotes P<0.001, ** denotes p<0.01, * denotes p < 0.05.*

***Pairwise correlations between self-reported scales and preference for more positive images***

In experiment 1, participants filled out five self-report questionnaires: the Life Orientation Test-Revised (LOT-R; Carver 2013), the anhedonia subscale from the Computerized Adaptive Test Personality Disorder Static Form (CAT-PD-SF, Simms et al. 2011), the Attitudes Toward Emotions Scale (Harmon-Jones et al. 2011), the anxiety subscale of the NEO PI-R (Costa and McCrae 1992), and the Social Phobia Inventory (SPIN, Connor et al. 2000).

None of the pairwise correlations between self-reported scales and preference for more positive images in any image category reached statistical significance (r between –0.341 and 0.233, all p>0.065). The full correlation matrix is presented in Table S4.

|  | Life Orientation Test-Revised | Anhedonia  (CAT-PD-SF) | Attitudes Toward Emotions Scale - Anger | Attitudes Toward Emotions Scale - Happy | Attitudes Toward Emotions Scale - Sad | Attitudes Toward Emotions Scale - Fear | Anxiety  (NEO PI-R) | Social Phobia Inventory |
| --- | --- | --- | --- | --- | --- | --- | --- | --- |
| IAPS | r = 0.202 | r = -0.24 | r = -0.221 | r = 0.163 | r = -0.293 | r = -0.333 | r = -0.201 | r = -0.043 |
|  | p = 0.285 | p = 0.202 | p = 0.241 | p = 0.388 | p = 0.116 | p = 0.073 | p = 0.288 | p = 0.823 |
| Food | r = -0.004 | r = -0.089 | r = -0.212 | r = 0.109 | r = -0.117 | r = -0.259 | r = 0.009 | r = 0.214 |
|  | p = 0.982 | p = 0.64 | p = 0.261 | p = 0.567 | p = 0.537 | p = 0.167 | p = 0.962 | p = 0.257 |
| Attractive | r = 0.095 | r = -0.213 | r = -0.198 | r = 0.16 | r = -0.026 | r = -0.151 | r = -0.129 | r = 0.233 |
|  | p = 0.617 | p = 0.259 | p = 0.295 | p = 0.398 | p = 0.891 | p = 0.425 | p = 0.498 | p = 0.216 |
| Happy | r = 0.133 | r = -0.239 | r = -0.084 | r = 0.114 | r = -0.254 | r = -0.205 | r = -0.123 | r = -0.243 |
|  | p = 0.483 | p = 0.203 | p = 0.66 | p = 0.55 | p = 0.176 | p = 0.277 | p = 0.517 | p = 0.196 |
| Angry | r = -0.002 | r = -0.039 | r = -0.172 | r = 0.091 | r = 0.03 | r = -0.201 | r = -0.029 | r = 0.198 |
|  | p = 0.994 | p = 0.838 | p = 0.363 | p = 0.633 | p = 0.875 | p = 0.287 | p = 0.877 | p = 0.294 |
| Fearful | r = -0.048 | r = -0.006 | r = -0.083 | r = -0.077 | r = -0.226 | r = -0.341 | r = -0.168 | r = 0.104 |
|  | p = 0.801 | p = 0.976 | p = 0.661 | p = 0.687 | p = 0.229 | p = 0.065 | p = 0.375 | p = 0.584 |
| Sad | r = -0.054 | r = 0.053 | r = -0.082 | r = 0.006 | r = -0.075 | r = -0.313 | r = -0.017 | r = 0.201 |
|  | p = 0.778 | p = 0.78 | p = 0.665 | p = 0.976 | p = 0.694 | p = 0.093 | p = 0.928 | p = 0.286 |
| Positive Preference | r = 0.046 | r = -0.12 | r = -0.184 | r = 0.098 | r = -0.158 | r = -0.309 | r = -0.094 | r = 0.128 |
|  | p = 0.809 | p = 0.527 | p = 0.33 | p = 0.606 | p = 0.406 | p = 0.097 | p = 0.622 | p = 0.501 |

*Table S4:* ***Pairwise correlations between self-reported scales and preference looking at more positive images for each stimulus category*** *– Each cell presents the correlation coefficient and p value of the correlation between a self-report questionnaire and positive preference in a certain category. None of the correlation is significant.*

***Differences between MDD and control participants in preference for more positive images in different categories***

| **Category** | **Control (N=22)** | **MDD (N=28)** | **t statistics** | **95% CI** | **d’** |
| --- | --- | --- | --- | --- | --- |
| IAPS | **M = 19.34, SD = 17.68** | **M = 4.16, SD = 13.18** | **t(48) = 3.48, p = 0.001** | **[6.41, 23.95]** | **0.97** |
| Food | M = 26.85, SD = 29.6 | M = 13.89, SD = 24.89 | t(48) = 1.68, p = 0.099 | [-2.54, 28.46] | 0.47 |
| Attractive | **M = 17.05, SD = 17.43** | **M = 6.78, SD = 16.9** | **t(48) = 2.11, p = 0.041** | **[0.46, 20.09]** | **0.59** |
| Happy | **M = 24.59, SD = 29.5** | **M = 4.81, SD = 13.3** | **t(48) = 4.13, p < 0.001** | **[10.14, 29.42]** | **0.86** |
| Angry | M = 11.95, SD = 26.02 | M = 3.81, SD = 16.56 | t(48) = 1.35, p = 0.185 | [-4.02, 20.3] | 0.37 |
| Fearful | M = 9.25, SD = 24.32 | M = 3.93, SD = 17.45 | t(48) = 0.9, p = 0.372 | [-6.56, 17.2] | 0.25 |
| Sad | M = 13.64, SD = 25.23 | M = 3.87, SD = 20.05 | t(48) = 1.53, p = 0.134 | [-3.1, 22.64] | 0.43 |

*Table S5:* ***Differences in preference for more positive images in different categories between MDD and control participants*** *– Each cell presents the results of two sample t-test comparing between the positivity preference between MDD and control participants. Significant results are in bold.*

***IAPS images numbers***

| Neutral | Positive |
| --- | --- |
| 5534.jpg | 1440.jpg |
| 5535.jpg | 1441.jpg |
| 5531.jpg | 1460.jpg |
| 5510.jpg | 1610.jpg |
| 7484.jpg | 1710.jpg |
| 5533.jpg | 1750.jpg |
| 7040.jpg | 1920.jpg |
| 2102.jpg | 2040.jpg |
| 2190.jpg | 2050.jpg |
| 2104.jpg | 2057.jpg |
| 2575.jpg | 2058.jpg |
| 2394.jpg | 2070.jpg |
| 2441.jpg | 2071.jpg |
| 2595.jpg | 2080.jpg |
| 2850.jpg | 2150.jpg |
| 2383.jpg | 2154.jpg |
| 2513.jpg | 2260.jpg |
| 2396.jpg | 2340.jpg |
| 2397.jpg | 2530.jpg |
| 2512.jpg | 2550.jpg |
| 5130.jpg | 5760.jpg |
| 2272.jpg | 5830.jpg |
| 2745.1.jpg | 5833.jpg |
| 7010.jpg | 5910.jpg |
| 9472.jpg | 8190.jpg |
| 2593.jpg | 8370.jpg |
| 2518.jpg | 8420.jpg |

*Table S6:* ***The full list of IAPS image numbers used in the experiment.***
